# Dissecting clonal hematopoiesis in the myeloid compartment of chronic lymphocytic leukemia and Richter transformation

**DOI:** 10.1002/hem3.70322

**Published:** 2026-02-16

**Authors:** Chiara Cosentino, Samir Mouhssine, Antonella Zucchetto, Ilaria Romano, Matin Salehi, Luca Vincenzo Cappelli, Fabio Iannelli, Mohammad Almasri, Nawar Maher, Lorenzo Fumagalli, Deborah Cardinali, Andrea Visentin, Jana Nabki, Luca Cividini, Bashar Al Deeban, Milena Lazzaro, Francesca Maiellaro, Annalisa Gaglio, Francesca Perutelli, Valentina Griggio, Riccardo Dondolin, Matteo Bellia, Maura Nicolosi, Silvia Rasi, Eleonora Secomandi, Valeria Caneparo, Abdurraouf Mokhtar Mahmoud, Clara Deambrogi, Sreekar Kogila, Joseph Ghanej, Mohammad Reshad Nawabi, Ilaria Del Giudice, Elisa Albi, Candida Vitale, Lydia Scarfò, Marta Coscia, Livio Trentin, Stefano Pileri, Paolo Ghia, Roberto Chiarle, Valter Gattei, Lodovico Terzi di Bergamo, Davide Rossi, Robin Foà, Gianluca Gaidano, Riccardo Moia

**Affiliations:** ^1^ Division of Hematology, Department of Translational Medicine Università del Piemonte Orientale and AOU Maggiore della Carità di Novara Novara Italy; ^2^ Clinical and Experimental Onco‐Hematology Unit Centro di Riferimento Oncologico di Aviano (CRO), IRCCS Aviano Italy; ^3^ Laboratory of Experimental Hematology Institute of Oncology Research Bellinzona Switzerland; ^4^ Hematology, Department of Translational and Precision Medicine “Sapienza” University Rome Italy; ^5^ Division of Hematopathology IEO European Institute of Oncology IRCCS Milano Italy; ^6^ Hematology Unit Azienda Ospedale Università Padova Italy; ^7^ Department of Medicine University of Padova Padova Italy; ^8^ A.O.U. Città della Salute e della Scienza di Torino e Dipartimento di Biotecnologie Molecolari e Scienze per la Salute Università di Torino Torino Italy; ^9^ Hematology Division A.O.U. Città della Salute e della Scienza di Torino Torino Italy; ^10^ Center for Translational Research on Autoimmune & Allergic Diseases – CAAD Università del Piemonte Orientale Novara Italy; ^11^ Division of Experimental Oncology IRCCS Ospedale San Raffaele Milano Italy; ^12^ Medical School Università Vita‐Salute San Raffaele Milano Italy; ^13^ Department of Medicine and Surgery University of Insubria Varese Italy; ^14^ Department of Pathology Children's Hospital and Harvard Medical School Boston Massachusetts United States; ^15^ Department of Molecular Biotechnology and Health Sciences University of Torino Torino Italy

## Abstract

The clinical and biological significance of clonal hematopoiesis (CH) has not been investigated in the myeloid compartment of chronic lymphocytic leukemia (CLL). By studying 488 newly diagnosed CLL through CAPP‐seq using a 28‐gene panel on granulocyte genomic DNA (gDNA), CH occurred in 231 (47.3%) patients. Cell sorting of cases that never developed Richter transformation (RT) confirmed that CH mutations, including CH‐related *TP53* mutations, were restricted to the myelomonocytic compartment and absent in CLL cells, as also documented by single‐cell DNA sequencing. CH associated with shorter overall survival (OS) (hazard ratio [HR] 1.36, 95% CI 1.04–1.77, P = 0.023); specifically, *TET2* mutations independently predicted inferior OS (HR 1.62, 95% CI 1.15–2.28, P = 0.01) after adjusting for age and for CLL‐related prognostic biomarkers, namely IGHV and *TP53* status. Regarding therapy‐related toxicities, CH correlated with a higher incidence of Grade ≥ 3 neutropenia (P = 0.004) after venetoclax‐based regimens. Sequential samples (*n* = 57) analysis showed that Bruton tyrosine kinase (BTK) and BCL2 inhibitors do not induce CH expansion, which was instead driven by chemotherapy. CH is significantly associated with a higher risk of second hematological malignancies only in chemo‐exposed patients. Single‐cell RNA sequencing of seven CH+ and six CH− CLL revealed that the T‐cell compartment of CH+ patients exhibits a less exhausted phenotype, documented by lower expression of TOX, the master regulator of T‐cell exhaustion, and a higher pro‐inflammatory profile. CH also influenced RT, since CH *ASXL1* mutations independently associated with higher RT risk (HR 11.19, 95% CI 4.09–30.62, P < 0.001). Overall, CH in CLL impacts survival, therapeutic toxicity, and transformation risk while also influencing the T‐cell immune compartment.

## INTRODUCTION

Clonal hematopoiesis (CH) is the clonal expansion of a hematopoietic stem cell and its progeny in otherwise healthy individuals, due to somatic driver mutations in genes mainly implicated in myeloid neoplasms.^1^, ^2^ CH prevalence rises with age and is linked to an increased overall mortality, higher risk of cardiovascular disease, and elevated risk of hematologic malignancies, especially of the myeloid lineage.^3^, ^4^, ^5^ In B‐cell malignancies, the presence of a CH clone may interact with the B‐cell neoplasm, potentially affecting outcomes and treatment tolerability.

Chronic lymphocytic leukemia (CLL), the most common adult leukemia,^6^ is a suitable model to study CH's role in disease outcome, evolution, and treatment. CLL mainly affects the elderly, where CH is more prevalent. CH may help explain the biological and clinical heterogeneity of CLL, ranging from indolent to aggressive forms like Richter transformation (RT). CLL is also associated with immune alterations, and CH affecting immunoregulatory genes may further influence immune cell activity in this leukemia.^7^

CLL treatment has shifted from chemoimmunotherapy to pathway inhibitors, mainly Bruton tyrosine kinase inhibitors (BTKi) and BCL2 inhibitors (BCL2i), making CLL a chemo‐free disease.^8^ This shift offers a new framework to study CH dynamics under chemo‐free versus chemo‐based treatments.^9^ Also, both CH and CLL pathway inhibitors have been linked to bone marrow dysfunction and cardiovascular complications, but the role of CH in predicting adverse events with BTKi and BCL2i remains largely unexplored.

To date, CH prevalence in CLL has been evaluated using genomic DNA from whole peripheral blood,^10^, ^11^ demonstrating that multiple CH mutations associate with reduced survival.^10^, ^11^, ^12^ However, whole peripheral blood in CLL mainly consists of neoplastic cells, potentially underestimating CH due to the disproportionate CLL‐to‐myeloid cell ratio. Additionally, mutations shared by both CLL and CH (e.g., *TP53* and *SF3B1*) cannot be clearly assigned to a specific entity or cell compartment in whole blood analysis.

On these grounds, the present study aimed to (i) selectively analyze CH in the myeloid compartment of a consecutive CLL cohort; (ii) carefully dissect CH and its consequences in the different peripheral blood compartments and at the single‐cell level; and (iii) evaluate the clinical impact of CH in terms of survival, RT, and therapy‐related toxicities.

## MATERIALS AND METHODS

### Patient characteristics

A cohort of 488 consecutive newly diagnosed CLL patients referred to our institution was assessed for CH in the myeloid compartment.^13^ Peripheral blood was collected and separated by gradient density using Ficoll Histopaque in all cases at the time of CLL diagnosis. In 57 cases, additional samples were obtained at sequential time points, namely at the time of treatment initiation and after therapy. The genomic DNA (gDNA) isolated from granulocytes represented the myeloid compartment for CH analysis. All patients were on active surveillance and attended at least two visits per year at our Hematology Division. Secondary malignancies and therapy‐related toxicities were documented using histopathological reports and/or visit records from our hospital databases, or, when necessary, by requesting relevant documentation from patients. The study was approved by the Ethical Committee of AOU Maggiore della Carità di Novara (study number CE 120/19). See also Supporting Information S16: Supplementary Appendix.

### CH analysis on gDNA from granulocytes by CAPP‐seq

gDNA from granulocytes, representing the myeloid compartment, was analyzed by CAPP‐seq using a panel of 28 genes frequently mutated in CH (Supporting Information S7: Table 1). A stringent bioinformatic pipeline for variant calling has been used as previously reported.^14^, ^15^ A variant allele frequency (VAF) threshold of 1% has been set for variant calling, and CH mutations were confirmed using the CH database that has been recently made available.^16^, ^17^ See also Supporting Information S16: Supplementary Appendix.

### Fluorescence‐activated cell sorting

The FACSAriaIII (BD Biosciences) instrument was used to sort the peripheral blood mononuclear cells (PBMCs) of 40 patients. CLL cells have been sorted using the CD19 and the CD5 antigens coupled with APC and BB515 fluorochromes, respectively. T cells were sorted using the CD3 antigen coupled to the PerCP fluorochromes. Monocytes have been sorted using the CD14 antigen coupled to the PE fluorochrome. DAPI (4′,6‐diamidino‐2‐phenylindole) was also used to select viable cells. The contamination of sorted populations was below 1%. gDNA extracted after cell sorting of the different compartments has been analyzed by the same 28‐gene CAPP‐seq panel previously described.

### Tapestri single‐cell DNA sequencing

Single‐cell DNA sequencing on viable frozen mononuclear cells was performed using the Tapestri platform (Mission Bio, CA). Three patients were analyzed on PBMCs during the CLL phase, and one patient was also analyzed on Richter cells isolated from ascitic fluid. See also Supporting Information S16: Supplementary Appendix.

### Single‐cell RNA sequencing

Single‐cell RNA sequencing (scRNA‐seq) was performed using the Singleron GEXSCOPE kit. The study was conducted on 13 CLL patients provided with frozen PBMCs. Out of the seven CH+ CLL patients, three harbored *DNMT3A* mutation, three *TET2* mutation, and one *ASXL1* mutation (Supporting Information S8: Table 2). The six CH− CLL patients used as controls were matched to CH+ samples for IGHV and *TP53* mutational status and for FISH karyotype (Supporting Information S8: Table 2). See also Supporting Information S16: Supplementary Appendix.

### Statistical analysis

Survival analysis starting from the time of CLL diagnosis for overall survival (OS), time to first treatment (TTFT), time to RT, and time to second cancer was performed by the Kaplan–Meier method and compared between strata using the log‐rank test. The adjusted association between exposure variables and events was estimated by multivariate Cox regression analysis. Mann–Whitney test for continuous variables and chi‐square test for categorical variables were used to compare patient characteristics and CH presence. False discovery rate (FDR) adjustment was used for multiple comparisons correction. Adverse events were collected according to the CTCAE v5.0. CH association with the development of second malignancies was evaluated using both Cox proportional hazards regression and cumulative incidence function analysis with Gray competing risk modeling, accounting for death as a competing event. See also Supporting Information S16: Supplementary Appendix.

## RESULTS

### Patient characteristics

**Figure 1:**
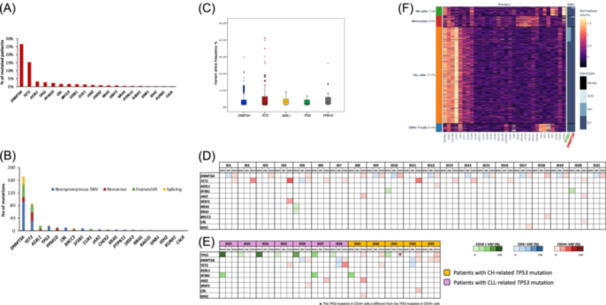
**Clonal hematopoiesis (CH) prevalence and correlation with chronic lymphocytic leukemia (CLL) baseline characteristics and disposition of CH mutations in different cell compartments**. **(A)** Bar charts representing the prevalence of the most frequent CH mutations (in red). **(B)** Bar charts representing the different types of CH mutations. **(C)** Boxplots representing the variant allele frequency of the different CH gene mutations. **(D)** CH mutations were identified in different cell compartments (i.e., CD19^+^/CD5^+^ CLL cells, CD14^+^ cells, and CD3^+^ cells) after cell sorting. The intensity of the color denotes the different variant allele frequency (VAF) of mutations according to different cell compartments, green for CD19^+^/CD5^+^ CLL cells, blue for CD3^+^ cells, and red for CD14^+^ cells. **(E)** CH mutations were identified in different cell compartments (i.e., CD19^+^/CD5^+^ CLL cells, CD14^+^ cells, and CD3^+^ cells) after cell sorting in patients harboring *TP53* mutations. Patients harboring CH‐related *TP53* mutations are highlighted in orange, and patients harboring CLL‐related *TP53* mutations are highlighted in purple. The intensity of the color denotes the different VAF of mutations (green for CD19^+^/CD5^+^ CLL cells, blue for CD3^+^ cells, and red for CD14^+^ cells). **(F)** Tapestri single‐cell DNA (scDNA)‐sequencing and immunophenotyping analysis of one CLL patient on peripheral blood. Each row corresponds to a single cell, and each column to a specific antibody. Cells are grouped according to genotype status, which is plotted on the right side of the slide (presence or absence of the variant, determined by zygosity). SNV, single nucleotide variant.

A total of 488 newly diagnosed and consecutively observed CLL cases composed the study cohort. The median age was 69.3 years (interquartile range [IQR] 61.7–76.6 years), and 210 (43.0%) were females. In line with other cohorts of unselected CLL, most patients were Binet A (*N* = 398, 84.0%), 312 (65.7%) presented mutated IGHV genes, and 43 (8.9%) harbored *TP53* disruption by mutation and/or deletion. The median follow‐up was 12.3 years. RT occurred in 24 (4.9%) patients. Complete patient characteristics are reported in Table 1 and Supporting Information S9: Table 3. Overall, the median survival was 12.4 years, and the median TTFT in Binet A CLL was not reached (12‐year treatment‐free probability of 64.6%) (Supporting Information S1: Figure 1).

**Table 1:** Patient characteristics.

Characteristics	Values
Gender, *n* (%)	
Male	278 (57.0%)
Female	210 (43.0%)
Age years (median)	69.3 (IQR 61.7–76.6)
Lymphocyte ×10^3^/µL (median)	8.9 (IQR 5.6–14.8)
Hb g/dL (median)	13.8 (IQR 12.8–14.9)
Platelet ×10^3^/µL (median)	204 (IQR 159–257)
Binet stage	
A	398 (84.0%)
B and C	76 (16.0%)
IGHV status	
IGHV unmutated	163 (34.3%)
IGHV mutated	312 (65.7%)
*TP53* disrupted	
Yes	43 (8.9%)
No	441 (91.1%)
Trisomy 12	
Yes	78 (16.9%)
No	384 (83.1%)
Del11q	
Yes	26 (5.6%)
No	436 (94.4%)
Del13q	
Yes	218 (47.2%)
No	244 (52.8%)
Richter transformation	
Yes	24 (4.9%)
No	464 (95.1%)
Treated	
No	306 (62.7%)
Yes	182 (37.3%)

Abbreviations: Hb, hemoglobin; IQR, interquartile range.

### CH is frequent in the myeloid compartment of CLL

A total of 358 mutations were detected in isolated granulocytes and considered as bona fide related to CH (Supporting Information S10: Table 4). Overall, at least 1 CH mutation was identified in 231 (47.3%) patients. The most frequent mutations identified on granulocytes affected *DNMT3A* in 129 (26.4%) patients, followed by *TET2* in 74 (15.2%), *ASXL1* in 15 (3.1%), *TP53* in 13 (2.7%), and *PPM1D* in 11 (2.3%). The other CH mutations analyzed were present in less than 2% of patients (Figure 1A).

**Figure 2:**
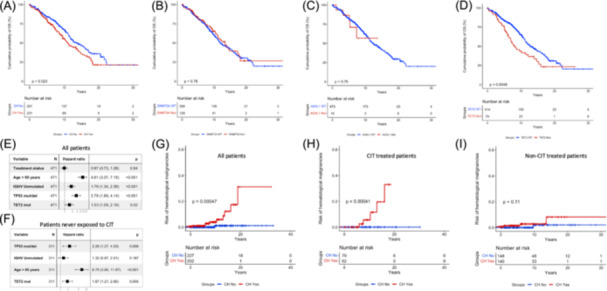
**Clinical impact of clonal hematopoiesis (CH) on overall survival and on risk of developing second malignancies**. **(A)** Kaplan–Meier estimates of overall survival (OS) according to the presence of any CH mutation. Patients with at least one CH variant are represented by the red curve, while patients without any CH variant are represented by the blue curve. Kaplan–Meier estimates of OS according to the presence of **(B)** *DNMT3A*, **(C)** *TET2*, and **(D)** *ASXL1* gene mutations. Mutated patients are represented by the red curve and wild‐type patients by the blue curve. **(E)** Forest plot multivariate analysis in terms of OS in all patients. **(F)** Forest plot multivariate analysis in terms of OS in patients who have never received therapy or patients who received only chemo‐free therapies. **(G)** Kaplan–Meier estimates of risk of second hematological malignancies excluding Richter transformation, stratified based on CH status. **(H)** Kaplan–Meier estimates of risk of second hematological malignancies excluding Richter transformation in patients who received prior chemoimmunotherapy (CIT), stratified based on CH status. **(I)** Kaplan–Meier estimates of second hematological malignancies excluding Richter transformation in patients never treated for CLL or who received only chemo‐free regimens, stratified based on CH status. In (G)–(I), patients with at least one CH variant are represented by the red curve, while patients without any CH variant are represented by the blue curve. P‐values are reported adjacent to the curves.

Mutations involved in both CLL and CH pathogenesis (e.g., *TP53*, *SF3B1*, *KRAS,* and *NRAS*) were classified as CH only when their VAF was higher in granulocytes than in matched PBMCs. When the VAF difference was less than 5%, CLL origin was confirmed by cell sorting and/or analysis of sequential PBMC samples with higher CLL lymphocyte counts. Mutations ascribed to CLL and not to CH are reported in Supporting Information S11: Table 5. To further assess whether these mutations originated from a common precursor already harboring CH, seven cases with mutations in genes involved in both CH and CLL pathogenesis and with higher VAF in PBMCs than in granulocytes were subjected to cell sorting and subsequent next‐generation sequencing (NGS) analysis. These mutations were detected exclusively in CLL cells and were absent in CD14⁺ and CD3⁺ cells (Supporting Information S2: Figure 2 and Supporting Information S12: Table 6). Therefore, at least in the context of the CLL cases analyzed, these mutations appear to be acquired during CLL development rather than originating from a common precursor already harboring CH.

The different types of CH mutations reflect CH biology. Most *DNMT3A* variants were nonsynonymous variants (52.3%), and the p.R882H hotspot was present in 9.3% (*N* = 12) of *DNMT3A‐*mutated patients. Most *TET2* and *ASXL1* variants were truncating events, accounting for 65.1% and 100% of cases, respectively (Figure 1B). The median VAF of the most frequently mutated genes was superimposable (all P > 0.1) among genes, being 2.29% for *DNMT3A*, 3.09% for *TET2*, 2.22% for *ASXL1*, 1.88% for *TP53*, and 2.70% for *PPM1D* (Figure 1C).

At CLL diagnosis, CH was significantly associated with older age, with 59.1% patients aged ≥75 years harboring CH. The median age of CH+ patients was 71.3 years compared to 67.2 years for wild‐type patients (P < 0.001).

### CH mutations distribute differently across the cell compartments of the CLL peripheral blood

To evaluate the distribution of CH mutations across different peripheral blood cell compartments, 33 CH+ patients that never developed RT were sorted by fluorescence‐activated cell sorting (FACS) and the CLL compartment (CD19^+^/CD5^+^ cells), the T‐cell compartment (CD3^+^ cells), and the myelomonocytic compartment (CD14^+^ cells) were independently sequenced (Supporting Information S13: Table 7). All CH mutations identified in CD14^+^ cells were also identified in the bulk granulocytes. Overall, the mutational analysis of CH in sorted cells showed that CH mutations are not present in the CLL compartment but, as expected, localize in the myelomonocytic compartment (i.e., CD14^+^ cells) (Figure 1D,E). In addition, *DNMT3A* mutations in 12 patients and *TET2* mutations in 1 patient were also detected in the T‐cell compartment, confirming the possible lympho‐monocyte skewage of some CH variants (Figure 1D,E).^18^

This analysis also allowed us to evaluate the distribution of *TP53* mutations in different cell compartments of CLL displaying CH (Figure 1E). In seven patients, *TP53* mutations were present uniquely in the CD19^+^/CD5^+^ CLL compartment and therefore could be assigned as true CLL‐related *TP53* mutations. Conversely, in five patients, *TP53* mutations were found exclusively in the myeloid compartment, therefore representing CH‐related *TP53* variants and not CLL‐related mutations (Figure 1E).

In addition, single‐cell DNA sequencing using the Tapestri platform was performed in one CH+ patient harboring the CH *DNMT3A* p.Y735C variant in the bulk myeloid compartment and the *SF3B1* p.H662D in the bulk CLL compartment. Single‐cell sequencing (total cells analyzed: 4428) confirmed that the CH *DNMT3A* mutation was absent in the CLL cell compartment, but present in monocytes and NK cells, reinforcing the notion of lympho‐monocyte skewing of *DNMT3A* variants related to CH.^18^ Conversely, single‐cell sequencing documented that the *SF3B1* mutation was uniquely present in CLL cells and absent in monocytes and NK cells (Figure 1F).

### CH‐related *TET2* mutations associate with shorter survival in treatment‐naïve CLL

Overall, the presence of at least one CH mutation is significantly associated with a shorter OS. The median survival for CH+ patients was 10.8 years compared to 13.6 years for CH− patients (P = 0.023) (Figure 2A). CH+ patients exhibiting a VAF between 1% and 1.99% had superimposable OS compared to CH+ patients harboring a VAF ≥ 2% (P = 0.914). By evaluating the three most frequent CH mutations independently, *DNMT3A* mutations (Figure 2B) and *ASXL1* mutations (Figure 2C) did not associate with a shorter survival (P = 0.778 and P = 0.753, respectively). Conversely, *TET2* CH mutations (Figure 2D) were significantly associated with a shorter survival, with a median OS of 7.9 years for *TET2‐*mutated patients compared to 12.7 years for wild‐type patients (P = 0.004, P = 0.008 after FDR correction). Importantly, *TET2* mutations retained an independent association with a shorter OS after multivariate analysis (hazard ratio [HR] 1.53, 95% CI 1.09–2.16, P = 0.02) when adjusted for treatment status (at least one line of CLL‐directed therapy), age, IGHV, and *TP53* status (Figure 2E). *TET2* mutations independently predicted shorter OS also when the analysis was restricted only to patients who had never received treatment or who had received only biological agents for CLL therapy (HR 1.87, 95% CI 1.21–2.90, P = 0.005) (Figure 2F).

**Figure 3:**
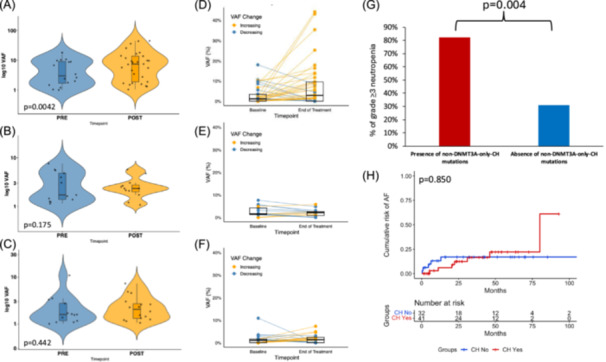
**Impact of chronic lymphocytic leukemia (CLL) therapies on clonal hematopoiesis (CH) expansion and clinical impact of CH mutations on therapy‐related toxicities with BCL2i and Bruton tyrosine kinase inhibitors (BTKi)**. **(A)** Violin plot representing the variant allele frequency (VAF) of CH mutations pre‐ and post‐chemoimmunotherapy. **(B)** Violin plot representing the VAF of CH mutations pre‐ and post‐BTKi exposure (median exposure: 37.6 months). **(C)** Violin plot representing the VAF of CH mutations pre‐ and post‐BCL2i. P‐values are represented adjacent to violin plots. **(D)** Box plot denoting VAF changes for individual patient pre‐ (in blue) and post‐chemoimmunotherapy (in yellow). Dots correspond to each CH mutation, and lines connect paired samples. **(E)** Box plot denoting VAF changes for individual patient pre‐ (in blue) and post‐BTKi (in yellow). Dots correspond to each CH mutation, and lines connect paired samples. **(F)** Box plot denoting VAF changes for individual patient pre‐ (in blue) and post‐BCL2i (in yellow). Dots correspond to each CH mutation, and lines connect paired samples. **(G)** Bar charts showing the prevalence of Grade ≥ 3 neutropenia occurring at any time during BCL2i therapy according to CH status. The % of Grade ≥ 3 neutropenia in patients with non‐*DNMT3A*‐only‐CH is represented by the red bar, and the % of Grade ≥ 3 neutropenia in patients without non‐*DNMT3A*‐only‐CH is represented by the blue bar. **(H)** Kaplan–Meier estimates of the risk of atrial fibrillation during BTKi according to the presence or absence of CH. Patients with at least one CH variant are represented by the red curve, and patients without any CH variant are represented by the blue curve. AF, atrial fibrillation.

To evaluate whether CH may predispose to a higher risk of CLL progression, TTFT was evaluated in 397 Binet A cases. The presence of at least one CH mutation in the myeloid compartment (12‐year treatment‐free probability of 65.8% for CH+ vs. 63.4% for CH− patients, P = 0.250) did not significantly associate with a shorter TTFT (Supporting Information S3: Figure 3A). Similarly, when CH mutations were tested individually, no significant association with a shorter TTFT was found for the presence of *DNMT3A* mutations (P = 0.290) (Supporting Information S3: Figure 3B), *TET2* mutations (P = 0.470) (Supporting Information S3: Figure 3C), or *ASXL1* mutations (P = 0.570) (Supporting Information S3: Figure 3D). Overall, these results suggest that CH does not boost the growth of treatment‐naïve CLL.

### The increased risk of second malignancies in CH+ CLL depends on previous treatment

**Figure 4:**
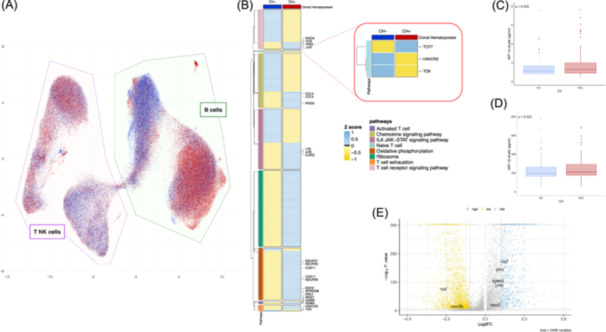
**Single‐cell RNA sequencing analysis in clonal hematopoiesis (CH)+ and CH− chronic lymphocytic leukemia (CLL) patients**. **(A)** UMAP dimensional reduction of B and T/NK cells pooled from all samples (*n* = 189,365 cells; seven CH+ and six CH− patients). Cells from CH+ patients are shown in red and cells from CH− patients in blue. Cell type and CH status allow us to distinguish four major clusters. A B‐cell cluster enriched in CH+ cells (bottom right of the figure), a B‐cell cluster enriched in CH− cells (top center of the figure), a T‐cell cluster enriched in CH+ cells (top left of the figure), and a T‐cell cluster enriched in CH− cells (bottom center of the figure). **(B)** Heatmap showing *Z*‐score–normalized expression of selected pathways in CH+ and CH− T cells across all samples. Genes were grouped by biological pathway, with key genes within each group labeled for clarity. Expression values were scaled using *Z*‐score normalization across all cells (upregulated genes in blue, downregulated in yellow), allowing comparison of relative expression patterns between CH− and CH+ T cells. The heatmap highlights pathway‐specific differences in gene expression with distinct transcriptional signatures observed between T‐cell populations according to CH status. **(C, D)** Boxplot showing plasma levels of MIP1α (CCL3) and MIP1β (CCL4), respectively, in CH+ (in red) and CH− (in blue) patients. **(E)** Volcano plot showing differential gene expression between T cells from CH+ and CH− patients. Upregulated genes (Log_2_FC > 1 and −Log_10_P‐value > 5) in CH+ T cells are highlighted in blue, while downregulated ones (Log_2_FC < −1 and −Log_10_P‐value > 5) are shown in yellow. Genes with Log_2_FC between −1 and 1 or a −Log_10_P‐value < 5 are considered not significant and represented in gray. Genes associated with the exhaustion and naïve T‐cell pathways are labeled.

**Figure 5:**
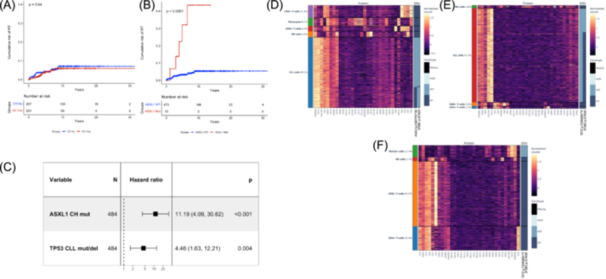
**Clinical impact and cell type distribution of clonal hematopoiesis (CH) in Richter transformation (RT)**. **(A)** Kaplan–Meier estimates of the risk of RT according to the presence of any CH mutation. Patients with at least one CH variant are represented by the red curve; patients without any CH variant are represented by the blue curve. **(B)** Kaplan–Meier estimates of the risk of RT according to the presence of *ASXL1* gene mutations. **(C)** Forest plot multivariate analysis in terms of time to RT. **(D)** Tapestri single‐cell DNA (scDNA)‐sequencing and immunophenotyping analysis of peripheral blood mononuclear cells (PBMCs) of one chronic lymphocytic leukemia (CLL) patient (RT1) that developed RT. **(E)** Tapestri scDNA‐sequencing and immunophenotyping analysis of PBMCs of a second CLL patient (RT2) who developed RT. **(F)** Tapestri scDNA‐sequencing and immunophenotyping analysis of Richter cells from the ascitic fluid of the RS2 patient. Each row corresponds to a single cell, and each column to a specific antibody. Cells are grouped according to genotype status, which is plotted on the right side of the slide (presence or absence of the variant, determined by zygosity). SNV, single nucleotide variant.

When considering hematological malignancies that developed after CLL diagnosis (excluding RT), the presence of CH predisposed to a higher risk of second hematological malignancies, that developed in nine CH+ patients and in only one CH− patient, accounting for a 12‐year risk of developing a second hematological malignancy of 5.5% for CH+ patients compared to 1.2% for CH− patients (P = 0.0004) (Figure 2G). The association of CH with a higher risk of developing second hematological malignancies retained significance also when considering death as a competing event (Supporting Information S4: Figure 4). The 9 second hematological malignancies were three myelodysplastic syndromes, three *JAK2‐*mutated myeloproliferative neoplasms, two mycosis fungoides, and one multiple myeloma. The only single second hematological malignancy that developed in a CH− patient was a smoldering myeloma. A significantly higher risk of developing second hematological malignancies in association with CH was only present in patients who had previously received chemoimmunotherapy (P = 0.0004) (Figure 2H), whereas it was not present in those who had never received treatment or who had received chemo‐free regimens as first‐line therapy (P = 0.11) (Figure 2I). Superimposable results have been obtained by excluding the non‐myeloid hematological malignancies, namely multiple myeloma and mycosis fungoides. All second hematological malignancies were confirmed by histopathology. Additionally, gDNA from four cases of secondary hematological malignancies (Ph‐negative myeloproliferative neoplasms) was analyzed using the same 28‐gene panel used for CH analysis. Compared with samples obtained at CLL diagnosis, all cases showed increased VAFs of pre‐existing CH mutations, and in some instances, the acquisition of additional mutations (Supporting Information S5: Figure 5).

### CLL pathway inhibitors do not lead to CH expansion, which is instead driven by chemotherapy

To evaluate whether different CLL therapies could impact CH growth, granulocytes from selected patients were analyzed before and after therapy (or during therapy for continuous BTKi). Among 25 treatment‐naïve patients treated subsequently with chemoimmunotherapy, 13 had at least one CH mutation before therapy exposure, with a median VAF of 1.13%. After chemoimmunotherapy, the number of patients with CH increased to 18, and the median VAF significantly rose to 2.85% (P = 0.0042) (Figure 3A,D and Supporting Information S6: Figure 6).

Conversely, in 17 patients treated with continuous BTKi (ibrutinib *N* = 15, acalabrutinib *N* = 1, and zanubrutinib *N* = 1) with a median exposure of 37.6 months, CH mutation VAF at baseline (1.53%) did not significantly increase at follow‐up (2.11%, P = 0.175), suggesting that BTKi do not promote CH growth (Figure 3B,E and Supporting Information S4: Figure 4). Similarly, among 15 patients receiving fixed‐duration venetoclax‐based therapy, the VAF of CH clones did not significantly increase after therapy (1.04% vs. 1.28%, respectively; P = 0.442), indicating that BCL2i also do not contribute to CH clonal expansion (Figure 3C,F and Supporting Information S4: Figure 4).

### CH predisposes to therapy‐related toxicities of pathway inhibitors

To evaluate whether CH predisposes to therapy‐related toxicities, CH was correlated with common adverse events of BCL2i and BTKi, namely, neutropenia and atrial fibrillation. Among 30 patients treated with venetoclax‐based therapy (14 with fixed‐duration venetoclax + anti‐CD20 in first line, 14 in second line, and 2 on continuous venetoclax post‐second line), non‐*DNMT3A*‐only‐CH mutations were significantly associated with a higher risk of Grade ≥ 3 neutropenia, occurring in 82.4% of patients with non‐*DNMT3A‐*only‐CH mutations versus 30.8% without (P = 0.004, P = 0.008 after FDR correction) (Figure 3G). Non‐*DNMT3A*‐only‐CH refers to patients with any CH mutations other than those with only a *DNMT3A* mutation.

The risk of atrial fibrillation was evaluated, starting from the initiation of BTKi, in 73 patients on continuous BTKi therapy (ibrutinib *N* = 61, acalabrutinib *N* = 7, and zanubrutinib *N* = 5) with a median exposure of 32.1 months. Atrial fibrillation developed in 12/73 (16.4%) patients. Overall, the presence of CH as a whole did not associate with a higher risk of atrial fibrillation, with a 24‐month risk of 12.4% for CH+ versus 17.0% for CH− patients (P = 0.850) (Figure 3H).

### CH affects the transcriptomic profile of T cells and associates with increased levels of pro‐inflammatory cytokines

Single‐cell RNA sequencing of seven CH+ and six CH− CLL (matched for IGHV and *TP53* status and FISH karyotype) revealed CH status‐driven differences in the transcriptional landscape of both B and T cells (Figure 4A). The complete list of pathways deregulated in B and T cells according to CH status is listed in Supporting Information S14: Table 8 and Supporting Information S15: Table 9. When focusing specifically on the T‐cell compartment, CH+ patients displayed cell pathways indicative of heightened T‐cell activation and a more pro‐inflammatory state, supported by increased expression of genes, including *JUN*, *FOS*, *RHOA*, *CCL3*, *CCL4*, *IL2RG*, and *LTB* (Figure 4B). Consistently, cytokine profiling of plasma samples (see Supporting Information S16: Supplementary Methods) from 244 patients collected at the time of CH assessment showed significantly elevated levels of CCL3 (MIP‐1α) and CCL4 (MIP‐1β) in CH+ CLL (P = 0.020 and P = 0.023, respectively) (Figure 4C,D), corroborating the predisposition toward a pro‐inflammatory state conferred by CH.

Furthermore, analysis of T‐cell exhaustion markers revealed that *TOX* (Figure 4B,E), the master regulator of T‐cell exhaustion induction, was significantly downregulated in T‐cells of CH+ patients. Concordantly, *TCF7* (Figure 4B), a critical transcription factor for maintaining multipotent progenitor T cells, was upregulated in CH+ CLL, suggesting that T‐cells of CH+ patients may exhibit a less exhausted phenotype.

### CH‐related mutations may affect the development of RT

The risk of RT conferred by different CH mutations at the time of CLL diagnosis was evaluated. Overall, in the present cohort, RT occurred in 24 patients (4.9%), a prevalence in line with other consecutive series of CLL.^19^, ^20^ Among these, 23 were clonally related diffuse large B‐cell lymphoma (DLBCL) variants, and 1 was a Hodgkin lymphoma variant. By considering the presence of any CH mutation, CH did not associate with a higher risk of RT (Figure 5A). Conversely, by evaluating the different CH mutated genes independently, *ASXL1* mutations showed a significant association with a higher risk of developing RT. At 12 years, the risk of RT was 43.5% in patients carrying *ASXL1* CH mutations compared to 5.4% for *ASXL1* wild‐type cases (Figure 5B). ASXL1 CH mutations (HR 11.2, 95% CI 4.09–30.62, P < 0.001) were independently associated with a higher risk of RT, even when adjusted for TP53 disruption harbored by the CLL clone (Figure 5C).

PBMCs of two patients, collected at the time of CLL diagnosis and who subsequently developed clonally related DLBCL, were subjected to single‐cell DNA sequencing using the Tapestri platform. In patient RT1 (total cells analyzed: 6559), the *ASXL1* p.C687Yfs*30 CH mutation identified in the granulocytes by bulk NGS was also identified by single‐cell DNA sequencing in the CLL compartment (Figure 5D). In addition, the CLL‐associated *SF3B1* p.G742D mutation, previously identified in the CLL compartment by bulk NGS, was confirmed in CLL cells by single‐cell analysis and was notably absent in the myeloid cells. In patient RT2 (total cells analyzed: 20,979), the *TET2* p.T1063Sfs*4 CH mutation identified in the granulocytes by bulk NGS was also present in the CLL cell compartment along with the *SF3B1* p.G742D mutation (Figure 5E). Single‐cell DNA sequencing of the Richter cells from the ascitic fluid of patient RT2 (total cells analyzed: 7861) also confirmed the presence of both *TET2* and *SF3B1* mutations in Richter cells, thus corroborating the clonal relationship between the CLL and RT phases (Figure 5F).

## DISCUSSION

This study addressed several clinical and biological questions on the role of CH in CLL, specifically investigating the patient's myeloid compartment. The results document that CH occurs in a sizeable fraction of CLL, influences the disease course by affecting survival and risk of RT, potentially contributes to therapy‐related toxicities during treatment with pathway inhibitors, and might warrant consideration for a proper diagnostic assignment of CLL molecular predictors (i.e., CLL‐related vs. CH‐related *TP53* mutations). Also, by influencing T‐cell activities, CH might potentially serve as a biomarker in the context of immunotherapies (i.e., CAR‐T cells and bispecific antibodies).

The prevalence of CH in our study, using a 1% VAF cut‐off, was 47.3%, slightly higher than previous reports in CLL patients.^10^, ^11^ This higher prevalence can be ascribed to greater sensitivity of CH assessment on granulocytes rather than whole peripheral blood, which in CLL is mostly composed of neoplastic B cells, diluting the CH clone and leading to CH underestimation. Consistently, the prevalence of CH on granulocytes at CLL diagnosis in the present study aligns with a recent analysis of the German CLL Study Group CLL14 trial that used a ≥0.5% VAF threshold and assessed CH after therapy, limiting myeloid contamination by CLL cells.^21^

To better dissect the relationship between CH and CLL, different peripheral blood compartments (CD19/CD5^+^ CLL cells, CD14^+^ cells, and CD3^+^ T cells) were analyzed after cell sorting and at the single‐cell level. CH was detectable in the sorted CD14^+^ compartment but not in the CLL population, indicating that, in CH+ patients, CLL does not derive from a cell already harboring CH or acquire CH progressively during leukemia progression. Conversely, in two patients who developed RT and were investigated by single‐cell DNA sequencing, the same CH mutations were also present in the CLL and Richter clones. We acknowledge that our sorting approach relied on peripheral blood and not on bone marrow cells, which also include hematopoietic stem and progenitor cells (HSPC). In another lymphoid malignancy, namely follicular lymphoma, CH mutations may be shared by lymphoma cells and HSPC.^22^ Larger studies are needed to clarify whether CLL transformation is promoted by CH‐related mutations in a precursor common to CLL and the myelomonocytic lineage.^23^, ^24^ Lastly, CH was also identified in T/NK cells, consistent with previous reports.^18^, ^25^, ^26^

Correlative analysis of CH with clinical behavior indicates that the myeloid compartment influences specific CLL outcomes. In early‐stage disease, CH does not enhance CLL growth, as shown by similar TTFT in Binet A CLL regardless of CH status. Conversely, consistent with reports linking CH to reduced survival,^3^, ^27^, ^28^ CH+ patients had shorter OS, mainly driven by *TET2* mutations, which remained prognostically significant after adjusting for age, IGHV, and *TP53* status. Notably, the impact of CH−related *TET2* mutations on OS is also evident in treatment‐naïve patients, suggesting that reduced survival is related to CH itself, independent of CLL therapy.

Consistent with previous reports,^10^, ^29^ we confirmed that chemoimmunotherapy significantly promotes clonal expansion of CH clones, which translates into a higher risk of second hematological malignancies. In contrast, the absence of CH expansion with BTKi and BCL2i aligns with a lack of increased risk of secondary hematological malignancies in CH+ CLL who never received chemoimmunotherapy. These findings, though limited by case numbers and follow‐up length, suggest that CH+ CLL patients receiving BTKi or BCL2i may have a lower risk of therapy‐related myeloid neoplasms than those treated with chemoimmunotherapy.

CH may also impact therapy‐related toxicities in CLL treated with pathway inhibitors. Neutropenia is the most frequent adverse event with venetoclax, and non‐*DNMT3A*‐only‐CH mutations (mostly *TET2* variants) are associated with a higher risk of Grade ≥ 3 neutropenia during BCL2i therapy. This may stem from the fact that *TET2* mutations impair granulocyte maturation,^30^ potentially leading to more severe neutropenia during venetoclax. This finding aligns with a previous report linking CH to prolonged cytopenia after venetoclax therapy^12^ and requires further validation in prospective trials with BCL2i.

From a diagnostic standpoint, the results of this study may bear implications for *TP53* mutational analysis in clinical practice. The recently updated ERIC guidelines acknowledge the relevance of low‐burden *TP53*‐mutated clones and no longer recommend a specific VAF cut‐off.^31^ However, the dissection of different cell compartments performed herein revealed that some *TP53* variants, especially at low VAF, do not derive from the CLL clone but from CH. Therefore, if CD19^+^ cell separation is not applied, low VAF CH‐related *TP53* variants may be mistakenly classified as CLL‐related. This finding is clinically relevant when choosing treatment, since the latest ESMO and NCCN guidelines indicate *TP53* disruption as a main decisional node.^8^, ^32^

In murine models, CH is associated with increased immune activity, likely due to reduced T‐cell exhaustion.^33^ Moreover, in B‐cell lymphomas treated with CAR‐T cells, CH links to a higher risk of T‐cell‐mediated toxicities such as cytokine release syndrome (CRS) and immune effector cell‐associated neurotoxicity syndrome (ICANS), as well as potentially greater therapeutic efficacy.^34^, ^35^ In this study, single‐cell transcriptomic analysis of T cells from CH+ CLL showed significant upregulation of pathways involved in T‐cell activation and proliferation. Our results align with murine findings in CLL patients,^33^ demonstrating that CH reduces T‐cell exhaustion by downregulating TOX, the master regulator of T‐cell exhaustion induction. In addition, compared to CH− cases, CH+ CLL also display a more pro‐inflammatory transcriptional signature of T cells and higher serum levels of pro‐inflammatory cytokines. These pro‐inflammatory features of CH may, in turn, influence the CLL microenvironment. With the growing use of CAR‐T cells and bispecific antibodies in CLL,^36^, ^37^ CH might serve as a biomarker to predict responses to these novel therapies.

This study is the first to selectively analyze the myeloid compartment of CLL patients, aiming at carefully unraveling the interplay between CH and CLL. The findings demonstrate that CH holds potential clinical relevance for both CLL and RT, and impacts on pathway inhibitor toxicity, while also modulating the T‐cell immune compartment of the patients with this leukemia.

## AUTHOR CONTRIBUTIONS

**Chiara Cosentino**: Methodology; data curation; software; formal analysis; writing—review and editing; conceptualization; writing—original draft. **Samir Mouhssine**: Conceptualization; methodology; software; formal analysis; data curation; writing—review and editing; writing—original draft. **Antonella Zucchetto**: Writing—review and editing; methodology; formal analysis. **Ilaria Romano**: Methodology; writing—review and editing; formal analysis. **Matin Salehi**: Methodology; writing—review and editing; software; formal analysis. **Luca Vincenzo Cappelli**: Methodology; visualization; software; formal analysis; writing—review and editing. **Fabio Iannelli**: Methodology; software; visualization; writing—review and editing; formal analysis. **Mohammad Almasri**: Writing—review and editing; formal analysis. **Nawar Maher**: Writing—review and editing; formal analysis. **Lorenzo Fumagalli**: Writing—review and editing; formal analysis; methodology; software. **Deborah Cardinali**: Methodology; formal analysis. **Andrea Visentin**: Writing—review and editing; formal analysis. **Jana Nabki**: Writing—review and editing; formal analysis. **Luca Cividini**: Writing—review and editing; formal analysis. **Bashar Al Deeban**: Writing—review and editing; formal analysis. **Milena Lazzaro**: Writing—review and editing; formal analysis. **Francesca Maiellaro**: Writing—review and editing; formal analysis. **Annalisa Gaglio**: Writing—review and editing; formal analysis. **Francesca Perutelli**: Writing—review and editing; formal analysis. **Valentina Griggio**: Writing—review and editing; formal analysis. **Riccardo Dondolin**: Writing—review and editing; formal analysis. **Matteo Bellia**: Writing—review and editing; formal analysis. **Maura Nicolosi**: Writing—review and editing; formal analysis. **Silvia Rasi**: Writing—review and editing; formal analysis. **Eleonora Secomandi**: Writing—review and editing; formal analysis. **Valeria Caneparo**: Writing—review and editing; formal analysis. **Abdurraouf Mokhtar Mahmoud**: Writing—review and editing; formal analysis. **Clara Deambrogi**: Writing—review and editing; formal analysis. **Sreekar Kogila**: Writing—review and editing; formal analysis. **Joseph Ghanej**: Writing—review and editing; formal analysis. **Mohammad Reshad Nawabi**: Writing—review and editing; formal analysis. **Ilaria Del Giudice**: Writing—review and editing; formal analysis. **Elisa Albi**: Writing—review and editing; formal analysis. **Candida Vitale**: Writing—review and editing; formal analysis. **Lydia Scarfò**: Writing—review and editing; formal analysis. **Marta Coscia**: Writing—review and editing; formal analysis. **Livio Trentin**: Writing—review and editing; formal analysis. **Stefano Pileri**: Writing—review and editing; formal analysis; supervision. **Paolo Ghia**: Writing—review and editing; formal analysis; supervision. **Roberto Chiarle**: Writing—review and editing; formal analysis; supervision. **Valter Gattei**: Supervision; formal analysis; writing—review and editing; methodology; software; conceptualization. **Lodovico Terzi di Bergamo**: Writing—review and editing; formal analysis; software. **Davide Rossi**: Writing—review and editing; supervision; formal analysis. **Robin Foà**: Supervision; resources; writing—review and editing; formal analysis; methodology; conceptualization. **Gianluca Gaidano**: Conceptualization; methodology; data curation; supervision; resources; funding acquisition; writing—original draft; writing—review and editing. **Riccardo Moia**: Conceptualization; methodology; data curation; supervision; project administration; formal analysis; visualization; investigation; writing—original draft; writing—review and editing.

## CONFLICT OF INTEREST STATEMENT

The authors declare no conflicts of interest.

## Supporting information

Supporting Information.

Supporting Information.

Supporting Information.

Supporting Information.

Supporting Information.

Supporting Information.

Supporting Information.

Supporting Information.

Supporting Information.

Supporting Information.

Supporting Information.

Supporting Information.

Supporting Information.

Supporting Information.

Supporting Information.

Supporting Information.

## Data Availability

The data that support the findings of this study are available from the corresponding author upon reasonable request and on the European Genome‐phenome Archive (EGA).
