# Supplementary figures and images for: Dissecting clonal hematopoiesis in the myeloid compartment of chronic lymphocytic leukemia and Richter transformation
Source: Hemasphere. 2026 Feb 16;10(2):e70322. doi: 10.1002/hem3.70322 (PMC12907972; doi:10.1002/hem3.70322)

**A**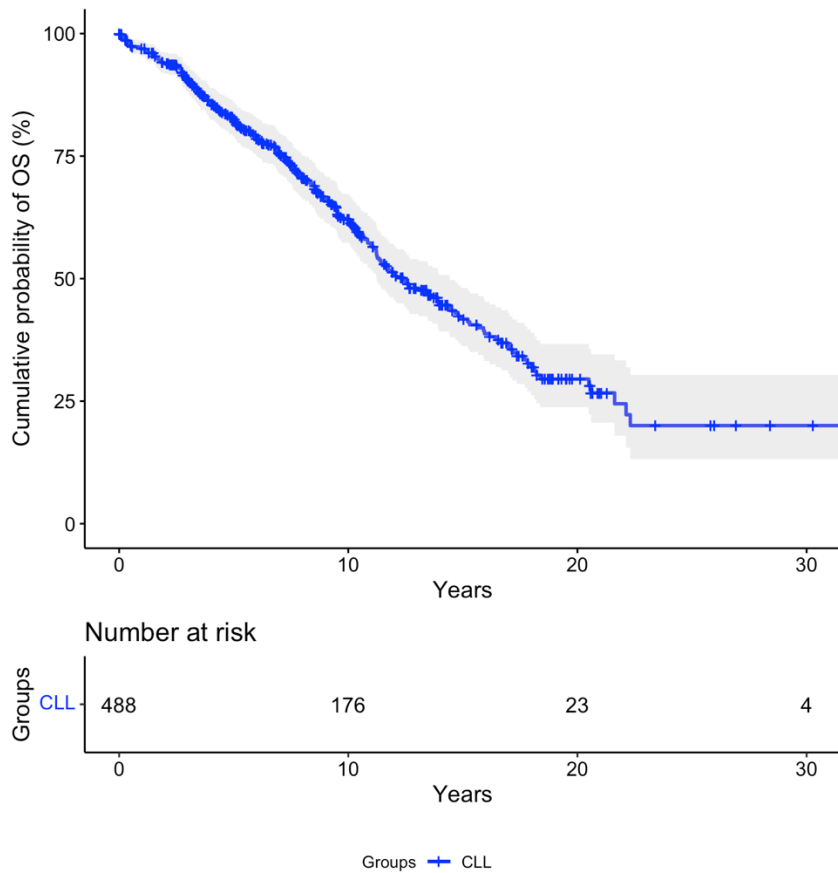**B**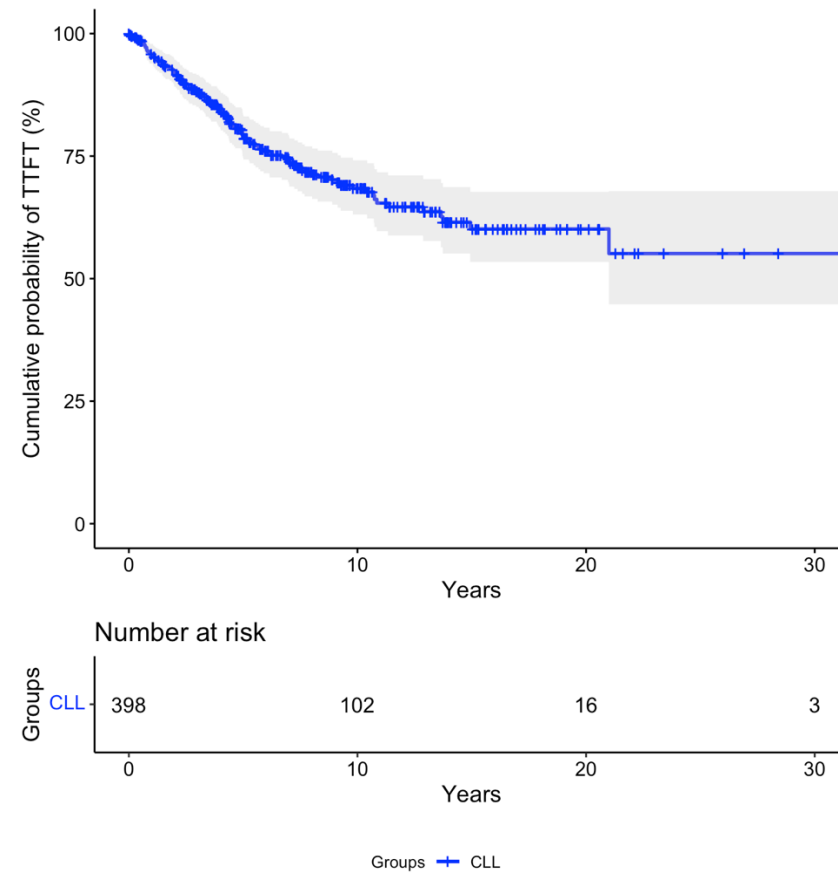**Figure S1**

Supplement: Supplementary file 1 — Supporting Information. [file HEM3-10-e70322-s012.pdf]

|              | ID34  |      |       | ID35  |      |       | ID36  |      |       | ID37  |      |       | ID38  |      |       | ID39  |      |       | ID40  |      |       |
|--------------|-------|------|-------|-------|------|-------|-------|------|-------|-------|------|-------|-------|------|-------|-------|------|-------|-------|------|-------|
|              | CD19+ | CD3+ | CD14+ |
| <i>TP53</i>  |       |      |       |       |      |       |       |      |       |       |      |       |       |      |       |       |      |       |       |      |       |
| <i>SF3B1</i> |       |      |       |       |      |       |       |      |       |       |      |       |       |      |       |       |      |       |       |      |       |
| <i>NRAS</i>  |       |      |       |       |      |       |       |      |       |       |      |       |       |      |       |       |      |       |       |      |       |
| <i>KRAS</i>  |       |      |       |       |      |       |       |      |       |       |      |       |       |      |       |       |      |       |       |      |       |

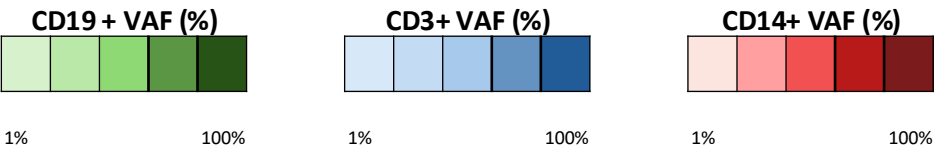

Figure S2

Supplement: Supplementary file 2 — Supporting Information. [file HEM3-10-e70322-s008.pdf]

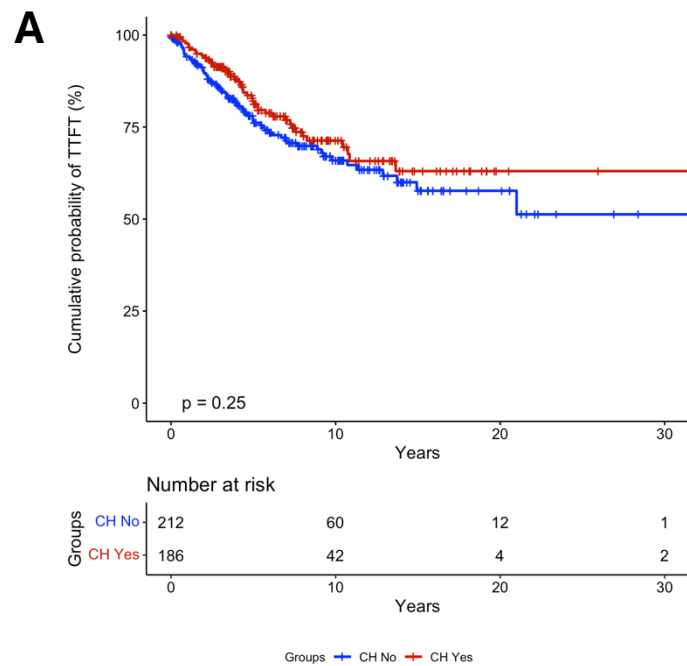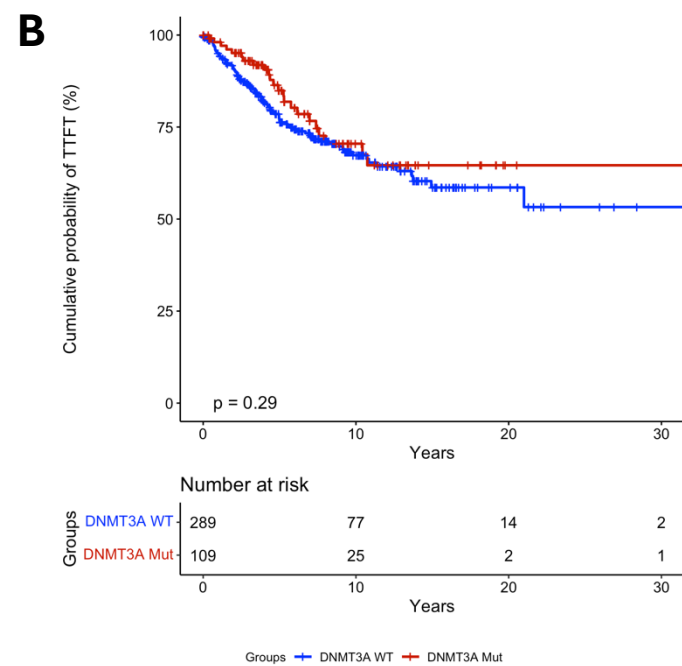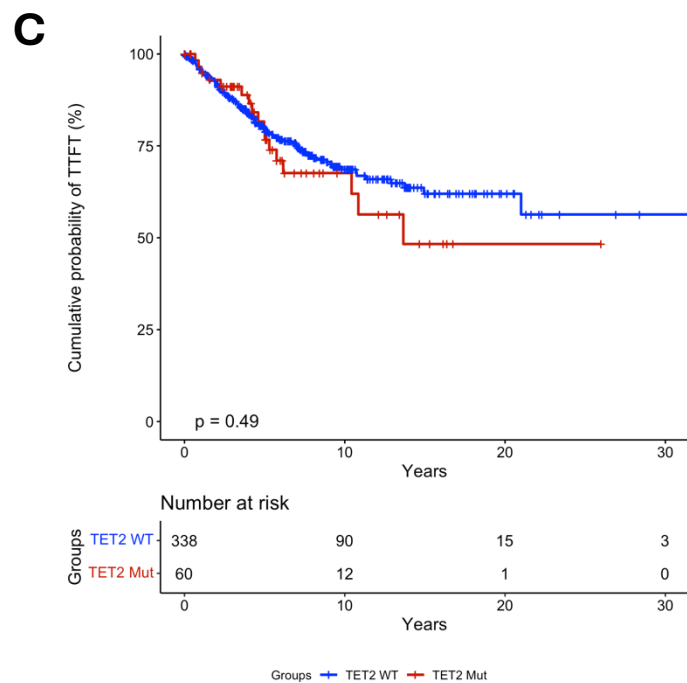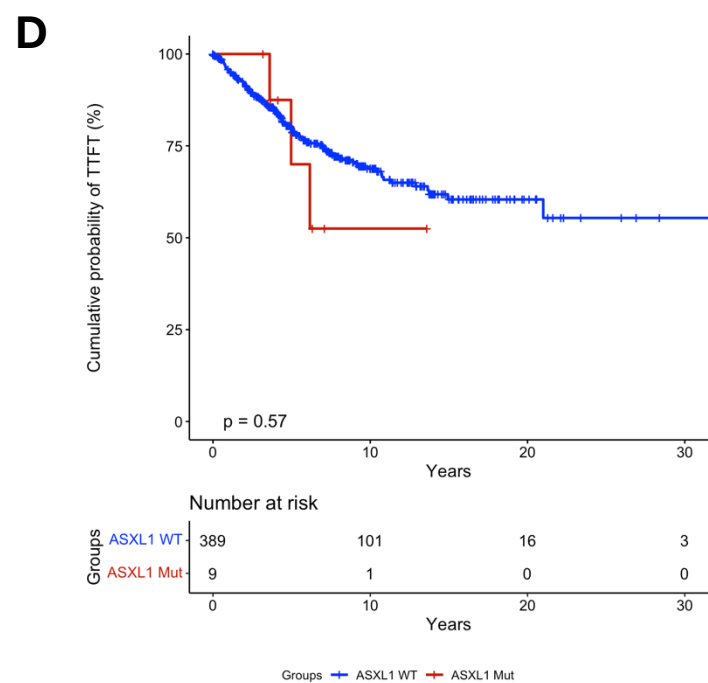

**Figure S3**

Supplement: Supplementary file 3 — Supporting Information. [file HEM3-10-e70322-s002.pdf]

**A**

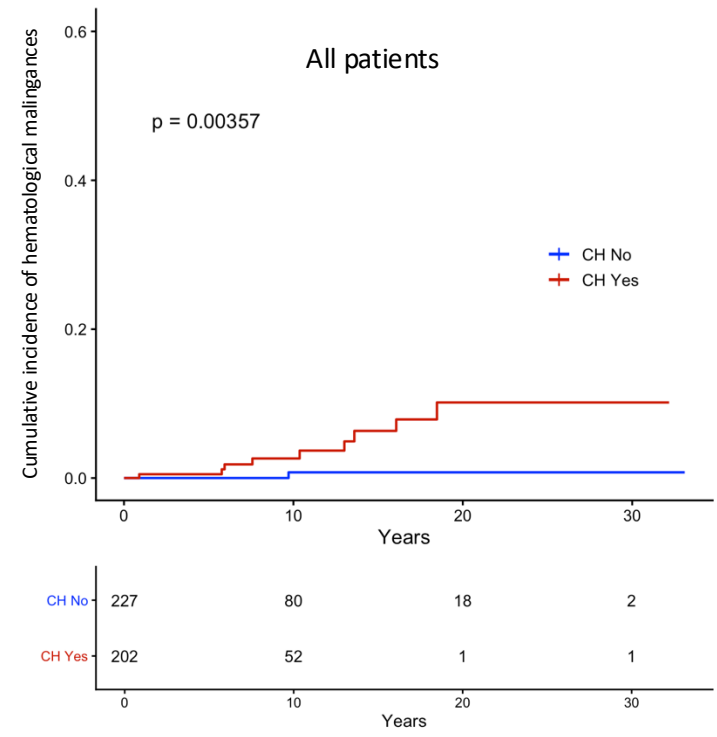

**B**

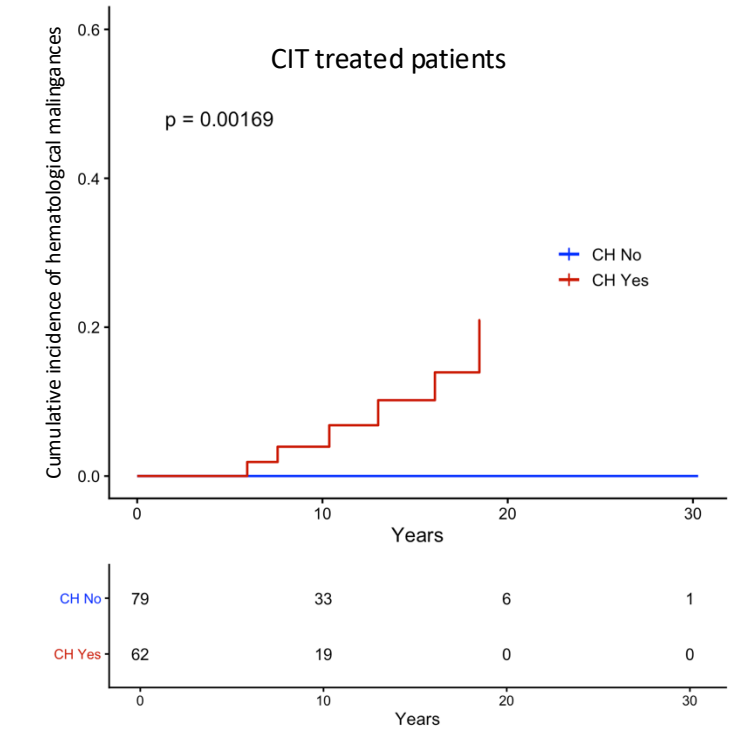

**C**

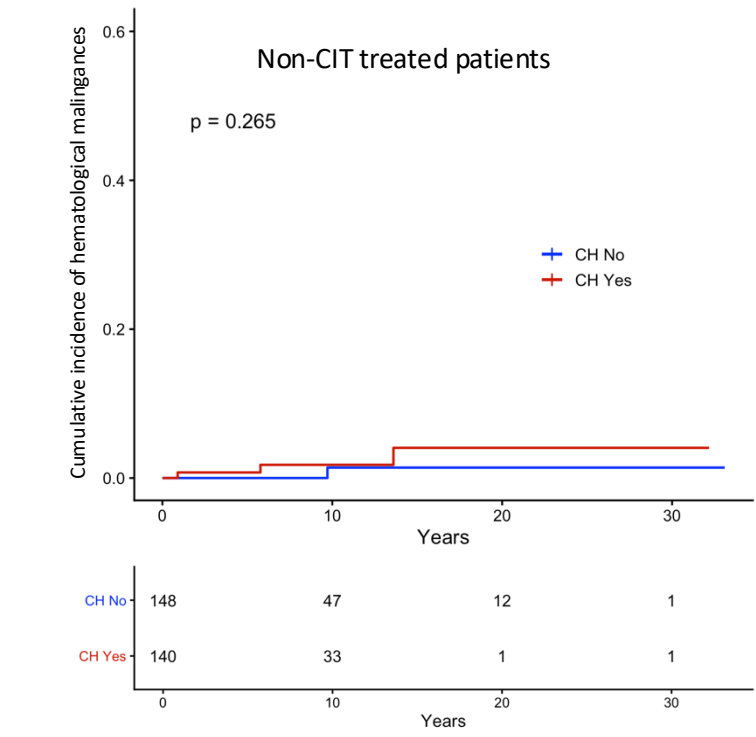

**Figure S4**

Supplement: Supplementary file 4 — Supporting Information. [file HEM3-10-e70322-s005.pdf]

**A**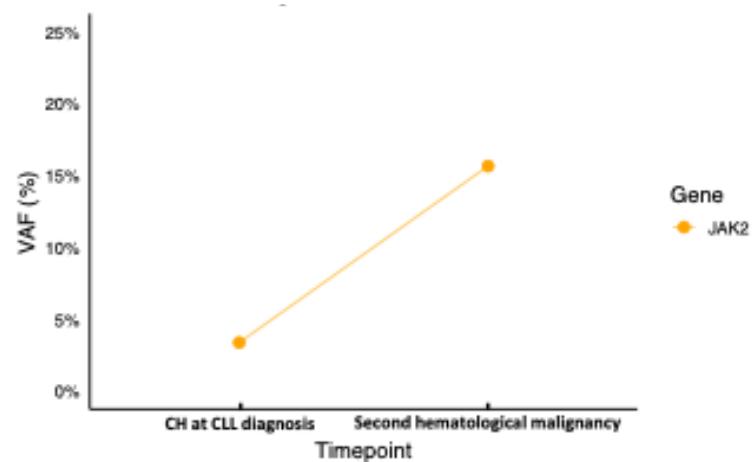**B**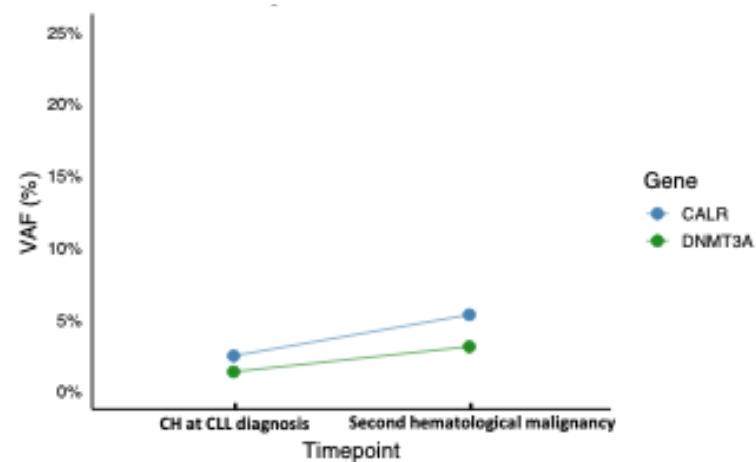**C**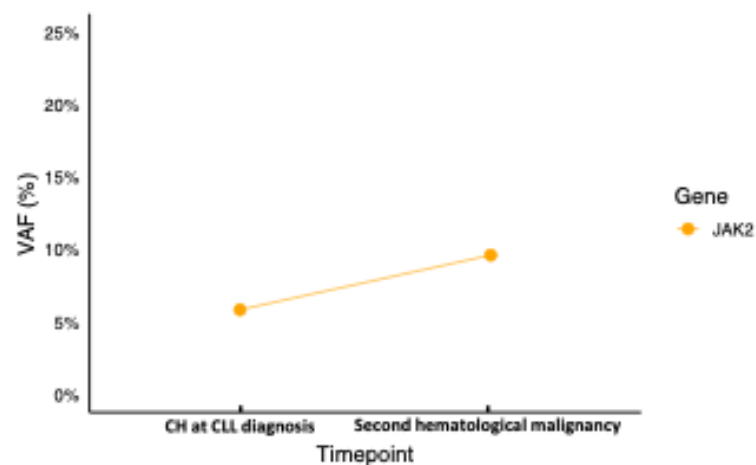**D**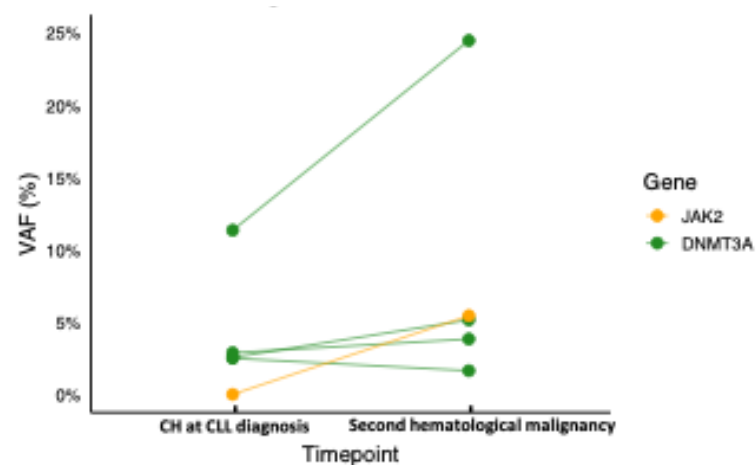**Figure S5**

Supplement: Supplementary file 5 — Supporting Information. [file HEM3-10-e70322-s011.pdf]

**A**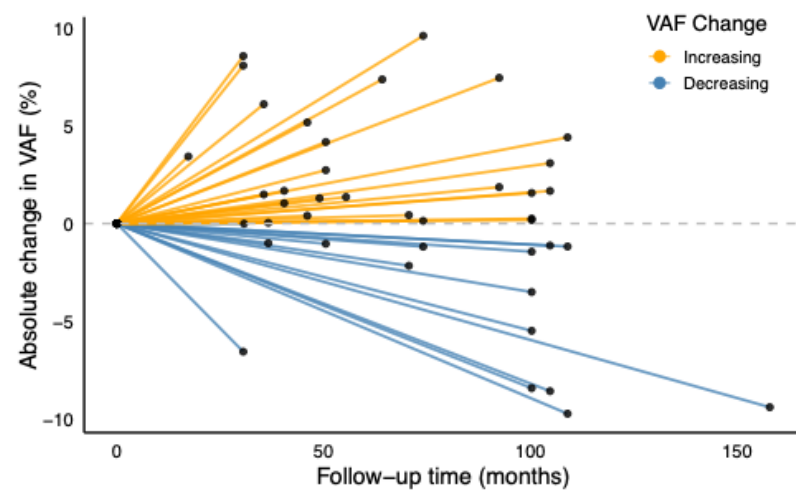**B**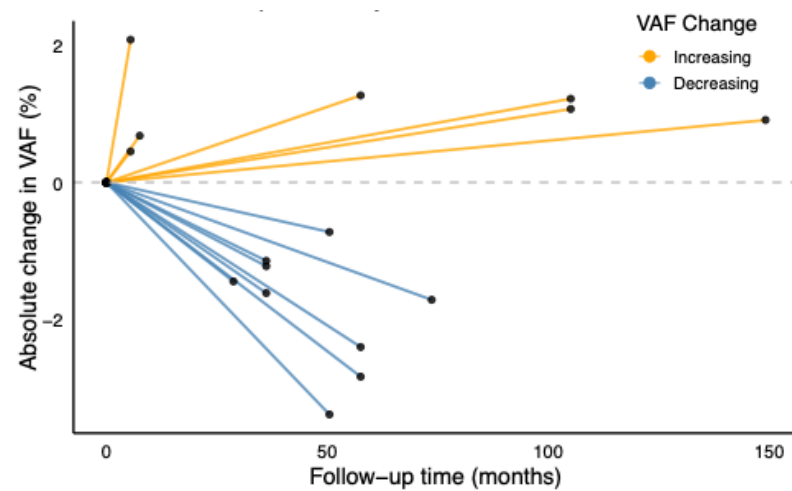**C**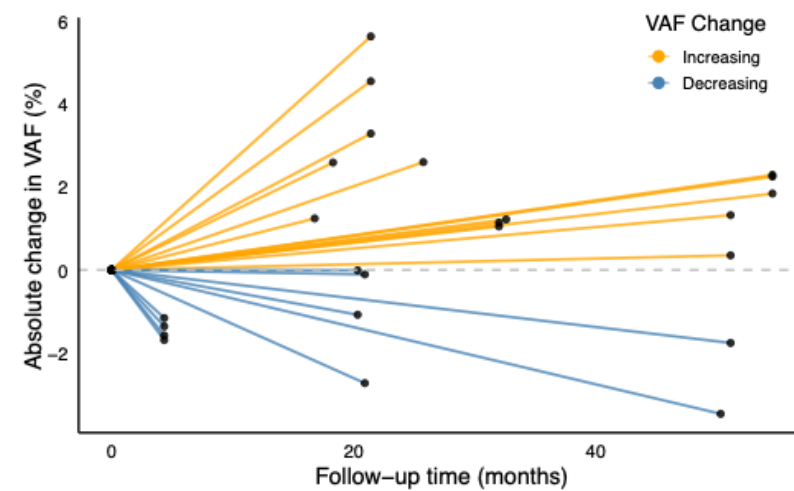**Figure S6**

Supplement: Supplementary file 6 — Supporting Information. [file HEM3-10-e70322-s016.pdf]
