# Supplementary material for: Dissecting clonal hematopoiesis in the myeloid compartment of chronic lymphocytic leukemia and Richter transformation
Source: Hemasphere. 2026 Feb 16;10(2):e70322. doi: 10.1002/hem3.70322 (PMC12907972; doi:10.1002/hem3.70322)
Supplement: Supplementary file 16 — Supporting Information. [file HEM3-10-e70322-s007.docx]

**Supplementary appendix**

**Supplementary methods**

**Sequential samples and patients evaluated for therapy-related toxicities**

To evaluate the evolution of CH dynamics during treatment, 57 patients provided with paired samples before and after therapy (chemoimmunotherapy, N=25; BTKi, N=17; BCL2i, N=15) were investigated. The association of CH with therapy-related toxicities was assessed in 103 CLL receiving pathway inhibitors, including 73 BTKi treated patients and 30 BCL2i treated patients.

**Separation of granulocytes from peripheral blood (PB)**

PB granulocytes were separated by Ficoll gradient density centrifugation as source of genomic DNA (gDNA). PB was diluted in 1:1 ratio with normal saline (NaCl 0.9%) and then centrifuged at 1800 revolutions per minute (rpm) for 25 minutes in a gradient differentiation Sigma DiagnosticTM Histopaque®-1077 Cell Separation Medium solution to obtain granulocytes and mononuclear cells (monocytes and lymphocytes).

**DNA extraction**

Granulocyte gDNA was extracted by using the “salting out” protocol.^1^ Cells were lysed with Lysis Buffer (Tris-HCl 1M, pH 8.2, NaCl 5M, 20 EDTA 0.5M), sodium dodecyl sulphate (SDS) 20% and digested with 20 mg/ml of proteinase enzyme (pronase E). Samples were incubated at 37°C overnight in a shaking incubator. Proteins were precipitated with 6M NaCl and subsequently discarded after centrifugation at 3200 rpm for 20 minutes. DNA was isolated by precipitation with pure ethanol and the resulting lactescent "jellyfish" of DNA was recovered with glass loops and washed three times in 75% ethanol. The excess of ethanol was evaporated, and the DNA was dissolved with TE Buffer (Tris-HCl 1M, pH 8.2, EDTA 0.5M).

**DNA quantification and fragmentation**

gDNA was quantified using the Quant-iTTM PicoGreen dsDNA Assay kit (ThermoFisher Scientific, Eugene, OR, USA). PicoGreen is a molecule that binds selectively to double helix DNA and allows to obtain a precise estimate of the amount of DNA. The fluorimetric reading was performed using the Infinite F200 fluorometer (TECAN, Männedorf, Switzerland) using the Magellan software. The fluorimetric readings were obtained at a wavelength of 485 nm in absorption and 530 nm in emission. For quantification, a standard curve was prepared using a DNA of known concentration and performing serial 1:2 scalar dilutions. Quant-iTTM PicoGreen dsDNA Assay kit was used at the 1:200 dilutions. The gDNA was fragmented by sonication with the M220 focused ultrasonicator (Covaris® Woburn, MA, USA) before library preparation in order to obtain 300-350 base pair fragments, representing the optimal length for analysis using the MiSeq and NextSeq 550 platforms (Illumina, San Diego, CA, USA). The size of the fragments was checked by using the 2100 Bioanalyzer Instrument with the High Sensitivity DNA kit (Agilent Technologies, St. Clara, CA, USA).

**Library design for hybrid selection**

A custom CAPP-seq gene panel, including coding exons and splice sites of 28 genes (target region: 29710 bp) that are recurrently mutated in CH, has been specifically designed for this project (Table S1).

**Library preparation and sequencing**

Libraries were generated using the KAPA HyperPrep kit (Roche Diagnostics, Pleasanton, CA, USA) and enrichment of regions of interest was achieved using a KAPA HyperChoice probe system (Roche Diagnostics, Pleasanton, CA, USA). Libraries were sequenced using 300-bp paired end runs on MiSeq Illumina platform and 150-bp end runs on NextSeq 550 Illumina platform. The library pool was denatured using 0.2N NaOH. An amount of 6 to 9.5 pM of denatured DNA (for the MiSeq platform) and 1.3 pM (for the NextSeq 550 platform) was loaded into the cartridge.

**Data analysis**

FASTQ sequencing reads were subjected to deduplication by using the FastUniq v1.1. Then, the deduplicated FASTQ sequencing reads were locally aligned to the GRCh37/hg19 version of the human genome assembly using the BWA v.0.6.1 software with the default setting, and sorted, indexed and assembled into a mpileup file using SAMtools v.1. Variant calling of single-nucleotide variants (SNVs), deletions and insertions (indel) at good quality nucleotide positions (Phred score >20) were carried out using the somatic function of the VarScan2 program. The variants called by VarScan 2 were annotated using wANNOVAR (https://wannovar.wglab.org/). Variants were annotated as single-nucleotide polymorphisms (SNPs) according to according to the National Center for Biotechnology Information (NCBI) database (https://www.ncbi.nlm.nih.gov/snp/), with the exception of *TP53* variants that were manually solved and scored as SNPs according to the International Agency for Research on Cancer TP53 database (http://p53.iarc.fr). Intronic variants, mapping >2 bp before the start or after the end of coding exons, and synonymous variants were then filtered out. Among the remaining variants, only protein truncating variants (i.e., indels, stop codons and splice site mutations), as well as missense variants not included in the dbSNP and annotated as somatic in the COSMIC v96 database (https://cancer.sanger.ac.uk/cosmic), were retained. All the variants were visualized using IGV (Integrative Genomics Viewer) software. Mutations of genes also involved in CLL pathogenesis^2^ and occurring at a higher VAF in the PBMCs (CLL compartment) compared to granulocytes were ascribed to CLL and not to CH.

**Bio-Plex Pro 27 Plex Human Cytokine Assay**

The Bio-Plex Pro 27 Plex Human Cytokine Assay (Bio-Rad Laboratories Ltd., Hercules, CA, USA) was used to simultaneously determine the plasma levels of 27 cytokines, chemokines and growth factors: fibroblast growth factor basic (FGF basic), eotaxin, granulocyte colony-stimulating factor (G-CSF), granulocyte‑macrophage colony-stimulating factor (GM-CSF), interferon (IFN)-γ, interleukin (IL)-1β, IL-1rα, IL-2, IL-4, IL-5, IL-6, IL-7, IL-8, IL-9, IL-10, IL-12, IL-13, IL-15, IL-17, IFN-γ-induced protein 10 (IP-10), monocyte chemotactic protein (MCP)-1, macrophage inflammatory protein (MIP)-1α and 1β, platelet-derived growth factor subunit B (PDGF-BB), regulated upon activation normal T cell expressed and secreted (RANTES), tumor necrosis factor (TNF)-α, vascular endothelial growth factor (VEGF). Data acquisition was performed using the Bio-Plex Reader 200 system.

Cytokines were measured in the plasma at the time of CLL diagnosis. Initially, serially diluted standards and diluted plasma samples (1:4 dilution) were prepared. These prepared solutions were then added to a microfilter plate pre-coated with antibody-coupled beads (50 μL) specific for each of the 27 cytokines. The plate was incubated at room temperature (RT) for 30 minutes with continuous shaking (850 ± 50 rpm) to facilitate the binding of cytokines to their respective beads. Following the initial incubation, the microfilter plate underwent three washing steps to remove unbound substances. Biotinylated detection antibodies (25 μL) were subsequently added to the plate and incubated with continuous shaking (850 ± 50 rpm for 30 minutes at RT), enhancing the detection specificity for each cytokine. After the detection antibody incubation, the plate was subjected to another set of three washing steps to eliminate excess detection antibodies. Streptavidin-Phycoerythrin (SA-PE) (50 μL) was then added to each well, and the plate was incubated at room temperature with shaking (850 ± 50 rpm for 10 minutes). This step is crucial as SA-PE binds to the biotinylated antibodies, allowing the detection of cytokines via fluorescent signals. Assay buffer (125 μL) was added to each well of the microfilter plate before proceeding with the read on a Bio-Plex 200 machine. Data acquisition was performed using the Bio-Plex Manager Software, with specific settings for bead count, sample size, and gating. Post-acquisition, the data were analyzed to remove outliers and ensure quality control. The obtained concentrations were compared to expected ranges to validate the assay performance​​.

**Tapestri single cell DNA sequencing**

Four viable frozen mononuclear cell samples from PB or from ascitic fluid were quantified by DeNovix CellDrop BF Cell Counters. One million viable cells were suspended in cell staining buffer at a concentration of 25,000 cells/μL and stained with the BioLegend Total-Seq D Heme Oncology Panel and Human TruStain FcX antibody (BioLegend, Cat. Nos. 399906, 422301), targeting 42 hematopoietic-specific proteins. Following staining, cells were washed in cell staining buffer and diluted to 2,800-3,200 cells/μL in cell buffer. Stained cells were encapsulated into microfluidic droplets using the Tapestri instrument, lysed, and barcoded for library preparation.

Library amplification, detailed in the table below, was carried out using a customized panel designed to target CH-related mutations identified in bulk granulocytes, as well as *bona fide* CLL-related mutations detected in bulk peripheral blood mononuclear cells.

| ***Gene*** | **Chr** | **Position** | **Ref** | **Var** | **Nucleotide Change** | **AA Change** |
| --- | --- | --- | --- | --- | --- | --- |
| ***ASXL1*** | chr20 | 31022572 | AGT | A | c.2058_2059del | p.C687Yfs*30 |
| ***DNMT3A*** | chr2 | 25463289 | T | C | c.A2204G | p.Y735C |
| ***SF3B1*** | chr2 | 198266611 | C | T | c.G2225A | p.G742D |
| ***SF3B1*** | chr2 | 198266611 | C | T | c.G2225A | p.G742D |
| ***SF3B1*** | chr2 | 198267373 | G | C | c.1984C>G | p.H662D |
| ***TET2*** | chr4 | 106158284 | TCA | T | c.3186_3187del | p.T1063Sfs*4 |

After emulsion breaking, libraries were purified using Ampure XP bead cleanup (Beckman Coulter). Protein libraries were enriched via biotinylated oligonucleotide bait pulldown. Separate libraries underwent PCR amplification using sequencing primers with library-specific indexes, followed by purification with Ampure XP beads.

The final libraries were quantified using a Qubit fluorometer (Thermo Fisher) and quality-checked on an Agilent 2100 Bioanalyzer (Agilent Technologies). Samples were pooled for sequencing with a 25% spike-in of PhiX and sequenced on a NextSeq 1000/2000 S4 flow cell (Illumina) to generate 150 bp paired-end reads.

FASTQ files were analyzed using the cloud-based Tapestri bioinformatics pipeline to perform adapter trimming, barcode correction, cell identification, read alignment to the human hg19 genome, and variant calling using GATK 4/Haplotypecaller. Informative variants were annotated, and cells were clustered based on their phenotypes. Filtering of the variants was performed with MissionBio’s Mosaic v3.4 package using the following settings: variants must have a minimum depth of 10 reads, minimum genotype quality of 30, and be called in a minimum of 50% of the cells. The genotypes and the cell matrix were consolidated into a h5 file for subsequent analysis using Python (version 3.8.19). Figures were generated using the Mission Bio Mosaic v3.4 pipeline.

**Single cell RNA sequencing**

Cryopreserved samples were thawed and checked for viability using trypan blue count. Viable cells were selected using EasySep™ Dead Cell Removal (Annexin V) (Stemcell TechnologiesTM). Samples with >85% viability were selected for single-cell RNA sequencing library preparation. In detail, 30,000∼35,000*cells were loaded into a NEO-chip SD (expected number of encapsulated cells: 10-15000) and scRNA-seq libraries were constructed according to the manufacturer’s instructions (Singleron GEXSCOPE Single Cell RNAseq Library Kit for NEO, Singleron Biotechnologies). Sequencing was performed on an Illumina Novaseq 6000 instrument with 150-bp paired-end reads. Reads QC, alignment, and gene expression quantification were performed using a Nextflow pipeline specifically designed for Singleron data (github.com/singleron-RD/scrna). Reads were mapped to the human genome (GRCh38/hg19).

Downstream analyses were performed mainly using the Seurat R package (v 5.2.1). Briefly, the proportion of mitochondrial RNA was calculated for each cell, and only those with mitochondrial content below 20% were included in downstream analyses. To minimize background signal, RNA contamination was estimated and removed using DecontX (v1.4.1).

Raw RNA counts were normalized with Seurat SCTransform function. Batch correction was performed using Harmony (v1.2.3). Principal component analysis (PCA) was performed on normalized data, and the top components were used for UMAP visualization.

Reference-based cell identity assignment was done using SingleR (v2.8.0). Both Monaco^3^ and Blueprint^4^ references were tested, with Blueprint, together with cell type-specific markers, selected for final annotation.

The main R libraries used to generate the figures presented in panel 4 are ComplexHeatmap (v2.22.0), EnhancedVolcano (v1.24.0) and circlize (v0.4.16).

**Statistical analysis**

Overall survival (OS) was defined as the time between the date of CLL diagnosis to the date of death (event) or last follow-up (censor). Time to first treatment (TTFT) was defined as the time between CLL diagnosis and start of first treatment because of CLL progression to symptomatic disease according to the iwCLL 2018 criteria.^5^ Survival analysis was performed by the Kaplan-Meier method and compared between strata using the Log-rank test. The adjusted association between exposure variables and events was estimated by Cox regression. Mann-Whitney test for continuous variables and Chi-square test for categorical variables were used to compare patient characteristics and CH presence. Adverse events were collected according to the CTCAE v5.0. CH association with the development of second malignancies was evaluated using both Cox proportional hazards regression and cumulative incidence function analysis with Gray competing risk modeling, accounting for death as a competing event. The analysis was performed with the Statistical Package for the Social Sciences (SPSS) software v.24.0 (Chicago, IL) and Rstudio Version 4.4.2, Inc.

**REFERENCES**

1. Miller SA, Dykes DD, Polesky HF. A simple salting out procedure for extracting DNA from human nucleated cells. *Nucleic Acids Res*. 1988;16(3):1215.

2. Knisbacher BA, Lin Z, Hahn CK, et al. Molecular map of chronic lymphocytic leukemia and its impact on outcome. *Nat Genet*. 2022;54(11):1664-1674.

3. Monaco G, Lee B, Xu W, et al. RNA-Seq Signatures Normalized by mRNA Abundance Allow Absolute Deconvolution of Human Immune Cell Types. *Cell Rep*. 2019;26(6):1627-1640.e1627.

4. Martens JH, Stunnenberg HG. BLUEPRINT: mapping human blood cell epigenomes. *Haematologica*. 2013;98(10):1487-1489.

5. Hallek M, Cheson BD, Catovsky D, et al. iwCLL guidelines for diagnosis, indications for treatment, response assessment, and supportive management of CLL. *Blood*. 2018;131(25):2745-2760.

**Supplementary figure legends**

**Figure S1: OS and TTFT of patients included in the study. (A)** Kaplan-Meir estimates for OS in the 488 CLL patients enrolled in the study. **(B)** Kaplan-Meir estimates for TTFT in the 398 Binet A CLL patients enrolled in the study.

**Figure S2:** **Mutations implicated in both CLL and CH pathogenesis that showed higher VAFs in PBMCs than in granulocytes were confirmed, after cell sorting, to originate from CLL rather than CH.** The intensity of the color denotes the different VAF of mutations.

**Figure S3: Clinical impact of CH in terms of TTFT in Binet A CLL. (A)** Kaplan-Meir estimates of TTFT according to the presence of any CH mutation. Patients with at least 1 CH variant are represented by the red curve, patient without any CH variant are represented by the blue curve. Kaplan-Meir estimates of TTFT according to the presence of **(B)** *DNMT3A*, **(C)** *TET2*, **(D)** *ASXL1* gene mutations. Mutated patients are represented by the red curve and wild type patients by the curve line. p-values are reported adjacent curves.

**Figure S4: Cumulative incidence of second hematological malignancies considering death as a competing event.** Cumulative incidence of second hematological malignancies excluding Richter transformation according CH status in **(A)** all evaluable cases, **(B)** in patients who received prior chemo-immunotherapy (CIT), and **(C)** in patients never treated for CLL or who received only chemo-free regiments. Patients with at least 1 CH variant are represented by the red curve, while patients without any CH variant are represented by the blue curve. The Gray p-values are reported adjacent to the curves.

**Figure S5. Connected dot plots of patients showing the different mutations identified as CH at the time of CLL diagnosis and mutations identified in the samples affected by second hematological malignancy**. Panel **A** denotes a patient who developed myelofibrosis. Panel **B** denotes a patient who developed essential thrombocythemia. Panel **C** denotes a patient who developed essential thrombocythemia. Panel **D** denotes a patient who developed polycythemia vera.

**Figure S6.** **CH analysis in sequential samples after chemo-immunotherapy** **(A), BTKi (B) and BCL2i (C)**. Scatter plots showing the absolute change in VAF according to time difference between baseline and sequential samples. The mutations with an increasing or decreasing VAF are indicated yellow and blue, respectively.
